# Holistic investigation of the anti‐wrinkle and repair efficacy of a facial cream enriched with C‐xyloside

**DOI:** 10.1111/jocd.16489

**Published:** 2024-08-06

**Authors:** Shanshan Zang, Juanjuan Chen, Cyril Chevalier, Ji Zhang, Shumei Li, Hequn Wang, Jing Li, Yangdong Chen, Hongling Xu, Le Sheng, Zhiming Zhang, Jie Qiu

**Affiliations:** ^1^ L' Oreal (China) Research and Innovation Center Shanghai China

**Keywords:** anti‐wrinkle/repairing/antiaging, C‐xyloside, in vitro, skin barrier, tape stripping

## Abstract

**Objective:**

To investigate the repairing and anti‐wrinkle efficacy of the facial cream enriched with C‐xyloside, aiming at comprehensively evaluating its skin anti‐ aging effect and clarify its potential mechanism of action.

**Methods:**

The repairing efficacy was studied on 3D epidermis skin model and the antiaging efficacy was studied on ex‐vivo human skin. Two clinical studies were conducted with Chinese females. In the first study, 49 subjects aged between 30 and 50 with wrinkle concerns were recruited and instructed to apply the investigational cream containing C‐xyloside for 8 weeks. Wrinkles attributes were assessed by dermatologist. Instrumental measurements on skin hydration, trans‐epidermal water loss (TEWL), and skin elasticity were also conducted. In the second study, 30 subjects aged between 25 and 60 with self‐declared sensitive skin and facial redness were recruited and instructed to apply the cream for 4 weeks. Biomarker analysis of the stratum corneum was conducted through facial tape strips.

**Results:**

The cream improved the histomorphology of the 3D epidermis skin model after SLS stimulation, and significantly increase the expression of LOR and FLG. On human skin, the cream improved the histopathology induced by UV, and significantly increased the protein content of COL I and COL III, collagen density and the number of Ki‐67 positive cell of skin compared with model group (*n* = 3, *p* < 0.01). The results from the first clinical study demonstrate a significant increased the skin hydration and elasticity by 21.90%, 13.08% (R2) and 12.30% (R5), respectively (*n* = 49, *p* < 0.05), and the TEWL values decreased by 33.94% (*n* = 49, *p* < 0.05), after 8 weeks application of the cream. In addition, the scores for nasolabial folds, glabellar wrinkle, underneath eye wrinkles, crow's feet wrinkle and forehead wrinkle in the volunteers exhibited a significant reduction of 34.02%, 43.34%, 50.03%, 33.64% and 55.81% respectively (*n* = 49, *p* < 0.05). The (rCE)/(fCE) ratio of volunteers based on tape stripping significant increased after using the sample cream (*n* = 30, *p* < 0.05).

**Conclusion:**

The cream containing C‐xyloside showed improvement of skin wrinkles and enhancement of skin barrier function. These efficacies may be attributed to the fact that the sample cream can increase the expression of skin barrier related proteins LOR and FLG, promote the maturation of cornified envelope, enhance collagen I and III protein expression and stimulate skin cell proliferation, to provide sufficient evidence supporting its antiaging efficacy of skin.

## 1 INTRODUCTION

The skin, as a vital barrier organ in the human body, inevitably undergoes an aging process under the combined influence of various stimulating factors. The skin is often seen as a static barrier that protects the body from external stimuli. Human skin is composed of three distinct layers: epidermis, dermis, and hypodermis, with varying degrees of specialization within each layer.^1^ Clinical hallmarks of aged skin include xerosis, wrinkles, melanocytic hyperplasia, telangiectasia, and diminished elasticity.^1^, ^2^, ^3^ The causes of wrinkle formation include loss of retinaculum cutis, thickening of the stratum corneum (SC), thinning of the dermis and epidermis and loss of collagen IV and VII at the dermal‐epidermal junction (DEJ).^4^

Elder skin is more likely to have damaged barrier with slower repair compared to younger skin. In addition, there are significant anatomical differences in basal TEWL, SC hydration, skin surface pH, and sebum content between aged and young skin. These defects are mainly due to the overall deficiency of all key epidermal lipids (especially cholesterol), focal reduction of the SC intercellular lamella, and decreased lamellar (LB) secretion.^5^ Therefore, repairing the epidermal barrier function is a crucial aspect of skin antiaging. Ultraviolet (UV) irradiation causes premature aging of the skin and gradual appearance of wrinkles. Due to the inability of telomeres to maintain their length during replication, the ability of primary fibroblasts to divide is limited and they are unable to proliferate, leading to premature and irreversible cell cycle arrest.^1^ Additionally, the loss of extracellular matrix (ECM) further accelerates wrinkle formation.^6^ From a histological perspective, the dermis is abundant in ECM components, including collagen, elastin, and glycosaminoglycans. Consequently, the emphasis of numerous antiaging studies has been predominantly on dermal aspects while lack of attention to the repairing of epidermal barrier function.^7^

C‐Xyloside, a synthetic C‐xylopyranoside derivative is as known C‐β‐D‐xylopyrano‐side‐2‐hydroxy‐propane, has been developed to simulate the activity of beta‐xyloside (O‐glycoside), increasing GAG synthesis by replacing the o‐glycoside position of the natural carbohydrate structure. Previous studies have shown that C‐xyloside stimulated increased glycosaminoglycan synthesis in human dermal fibroblasts, mainly manifested by an increase in the chondroitin/dermatan sulfate ratio, suggesting the potential and application of this compound in skin repair/regeneration.^8^ C‐xyloside can improve the ultrastructure of the DEJ by significantly increasing the expression of ɑ6‐integrin and laminin‐332, and promote the expression of CD44 in the epidermis.^9^ Clinical studies have demonstrated that C‐xyloside can significantly improve facial wrinkles, radiance, complexion evenness, and skin firmness in subjects.^10^

While these studies have provided compelling evidence supporting the efficacy of C‐xyloside in enhancing skin quality and slowing down the skin aging process, our understanding of its mechanism of action (MOA) remains limited. In this study, a cream mainly containing C‐xyloside as the active substance were formulated, which was composed of water, glycerin, propylene glycol, pentanediol, xanthan gum, sodium polyacrylate, butyrospermum parkii (shea) butter, and myristyl myristate. The anti‐wrinkle and repairing efficacy of C‐xyloside cream was investigated from multiple dimensions with 3D human epidermal skin model,^11^ ex‐vivo human skin, and clinical trials to comprehensively evaluate its antiaging efficacy and the MOA, to enrich the existing knowledge on C‐xyloside as an essential skin antiaging ingredient.

## 2 MATERIALS AND METHODS

### 2.1 Treatment on reconstructed 3D human epidermis model

Reconstructed 3D human epidermis model (Biocell, China) experiments were conducted using a control group, model group (0.1% SLS), positive control group (0.1% SLS and 50 μM WY14643 [A potent PPARα agonist, Sigma, USA]), and experimental group (0.1% SLS and 12.5 μL cream (L'Oréal, China)). The skin models of all four groups mentioned above were cultured in an incubator with 5% CO_2_ at 37°C for 24 h then the skin was fixed for 24 h in 4% paraformaldehyde (Beyutime, China). After washed with PBS, the fixed skin samples were dehydrated by step wise immersion in solutions of increasing ethanol concentration, embedded in paraffin overnight, then were sealed with dry gum and sliced into sections.

Hematoxylin and eosin (HE) (Beyutime, China) staining were performed on skin sections, the contents of loricrin (LOR) and filaggrin (FLG) were detected by immunofluorescence staining (Abcam, UK). Images were obtained and analyzed by microscope (Olympus, CKX41, Japan) and fluorescence microscopy (Leica; DM2500, Germany).

### 2.2 Experiment on ex‐vivo human skin

The ex‐vivo human skin was donated by volunteers who have signed an informed consent form. Biopsy materials were obtained in accordance with Chinese law and in compliance with the requirements of Local Ethics Committees. The ex‐vivo skin (three females) was cut into small discs with a diameter of 0.6 cm, cultured with DMEM/F‐12 medium supplemented with 10% fetal bovine serum, 100 units/mL penicillin, and 100 μg/mL streptomycin. The skin incubated in a humidified incubator with an atmosphere of 95% air and 5% CO2 at 37°C for 48 h with the epidermis facing upward, followed by UV irradiation and cream treatment.

The skin was conducted using a control group, model group (UV irradiation), positive control group (UV irradiation, vitamin C (VC) 100 μg/mL and vitamin E (VE) 7 μg/mL), and experimental group (UV irradiation and 2 μL sample cream). The positive control was added into the culture medium and the cream was applied on the surface of skin once a day for 7 days. UV irradiation was irradiated at the dose 30 J/cm^2^ UVA and 50 mJ/cm^2^ UVB for 4 days once a day and the skin treatment with the cream after UV irradiation, the residual cream should be removed from the skin before UV irradiation. Then the skin treated with cream for another 3 days as described above without UV irradiation. The model of all four of the above‐mentioned groups were cultured in an incubator with 5% CO_2_ at 37°C. After treatment the skin were collected, the collagen density was measured with an ultrasonic skin tester (DermaLab Combo, Denmark). The rest of the skin were fixed for 24 h in 4% paraformaldehyde. After fixed samples were washed with PBS and dehydrated by step wise immersion in solutions of increasing ethanol concentration, embedded in paraffin overnight, then were sealed with dry gum and sliced into sections.

Sections were stained with HE, Immunohistochemical and immunofluorescence staining (Abcam, UK) of the contents of Ki‐67, Collagen I (COL I) and Collagen III (COL III) were performed. Images were obtained and analyzed by microscope.

### 2.3 Clinical evaluation on antiaging efficacy

A mono‐centric, single‐blind clinical study was conducted in China, between June to August in 2022. This study strictly followed the guidelines of the declaration of Helsinki. Volunteers gave their informed consent to participate in the study. The study was approved by the local ethic committee (ethic approval SECCR2021‐207‐02).

Forty‐nine Chinese women, all skin types, aged 30–50 were recruited, 50% of them self‐declared as having sensitive skin. They all presented with skin qualities adjusted by dermatologist based on Skin Aging Atlas.^12^ forehead wrinkles (2 ≤ grade <5, in Atlas); underneath eye wrinkles (2 < grade ≤6, in Atlas); crow's feet wrinkles (grade ≥2, in Atlas), Pores (grade >1, Dermascore, in Atlas); skin smoothness (3 ≤ grade ≤6, Griffith 10‐points, to 9 scale); skin elasticity (3 ≤ grade ≤6, Griffith 10‐points, 0–9 scale). Subjects with a skin disease in the test areas as well as skin allergy (particularly e.g., acne, rosacea, eczema) were excluded, subjects with history of medical beauty treatment and taking part in antiaging studies in the last 3 months before study were excluded.

Subjects were asked to apply the cream twice a day (morning and evening), after standard facial cleansing. The standard sunscreen is applied to face every morning 15 min before sun exposure. The subjects had images taken, dermatologist assessment and instrumental measurement at the time points 0 week (baseline), 2 week (2 W), 4 week (4 W), and 8 week (8 W). They are acclimatized to ambient conditions for 30 min before images taken, dermatologist and instrument assessment. The moisture content of the skin was measured by Corneometer (Courage & Khazaka, Germany), the transepidermal water loss (TEWL) was measured by Vapometer (Delfin Technologies, Finland), skin's elastic properties was measured by Cutometer® Dual MPA 580 (Courage + Khazaka, Germany). The facial photo is captured by VISIA‐7 (Canfield, New Jersey) on the front, left and right sides with all lighting modes.

### 2.4 Evaluation of repairing efficacy of based on volunteers by tape stripping

Thirty Chinese women who met the inclusion criteria, aged between 25 and 60 years old, were recruited and those who self‐declared sensitive skin and significant redness of the face could be observed. All subjects signed informed consent prior to the study and agreed to comply with all requirements outlined in the study protocol. The study was approved by the Internal Ethic Committee (BXLL2022036).

In addition to standard skincare products, the volunteers applied the sample cream once in the morning and once in the evening after cleaned their face during the testing. After the subject's face was cleansed and acclimated in a controlled environment with constant temperature and humidity for 20 min, the sampling tape was applied on both sides of the left and right cheeks respectively prior to product application. Subsequently, SC was obtained by gently pressing the tape for several seconds before product usage.^13^ The volunteers were instructed to apply the sample cream twice daily, in the morning and evening. Following a continuous usage period of 28 days, tape stripping was employed to collect SC from the volunteers' post‐application after washing their face.

Tape stripped SC was cut into small pieces and immersed in 1 mL of dissociation buffer, followed by boiling at 100°C for 10 min. The dissociation buffer was centrifuged at 4000*g* for 10 min, and the CEs were obtained by collecting precipitation. The CEs fixed with paraformaldehyde, then incubated with anti‐involucrin antibody. The primary antibody allowed to react at 4°C overnight, then FITC‐labeled secondary antibody was applied. After extensive washing with PBS, CEs were stained with Nile red for lipids. Fluorescence signals of involucrin (IVL) and lipids were observed with fluorescence microscope and images were obtained.^14^

### 2.5 Statistical analysis

Statistical analysis and mapping were performed using GraphPad Prism 8.02. The data of in vitro experiment were expressed as Mean ± SD through *t*‐test analysis. The results of the clinical trial were presented as Mean ± SEM. If the data is under normal distribution, one‐way ANOVA will be used, otherwise, Wilcoxon Signed Rank Test will be used. Significant level *α* = 0.05.

## 3 RESULTS

### 3.1 Protective effects of investigational cream on skin barrier damage as assessed in SLS‐induced 3D human epidermis model

**FIGURE 1:**
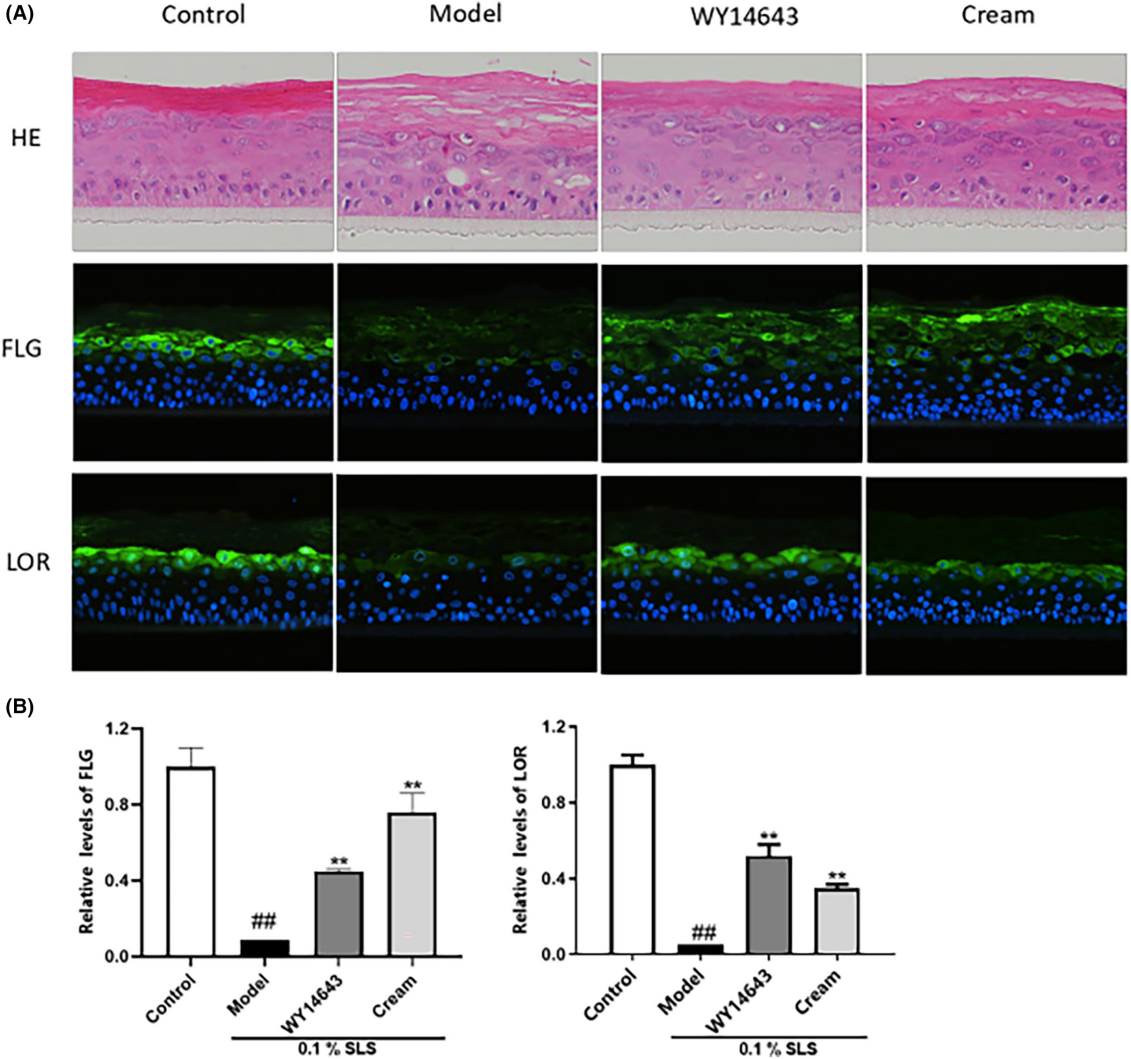
Effects of sample cream treatment on SLS‐induced skin model as assessed via HE staining and immunofluorescence staining. HE: The red stained areas correspond to keratin, and the blue‐purple stained areas correspond to the nucleus. WY14643 (50 μM) was used as the positive control. The vacuolation, reduction of living cells, and damage to the stratum corneum induced by SLS stimulation were significantly ameliorated upon treatment with both the positive control and the sample cream. FLG, LOR: The blue‐stained regions correspond to the nucleus, while the yellow and green‐stained regions correspond to the cell membrane. (A) The Histomorphological changes and expression of FLG, LOR in skin model was analyzed under HE staining and fluorescence microscope after treatment with the cream containing C‐xyloside for 24 h. (B) Relative expression levels of FLG, LOR were significantly increased after sample cream treatment compared with the model group (0.1% SLS). Values are expressed as means ± SD (*n* = 3). ^###^ *p* < 0.01 vs. the control group; **p < 0.01 vs. the model group.

Histomorphological changes observed via HE staining analysis demonstrated that in the model group (0.1% SLS induced), obvious damage appeared as compared with the control group, the SC of the epidermal layer was loose and thickened, vacuoles appeared in the living cell layer. (Figure 1A). SLS exhibits potent cleansing efficacy and is widely used as a detergent in cosmetic formulations. However, excessive concentrations of SLS can compromise the integrity of the skin barrier and induce skin irritation.^15^ It was also confirmed by the skin model in this study.

It has been reported that the expression of upper spinous/granular layer structural proteins (IVL, profilaggrin, filaggrin, and loricrin) increased after topical treatment with PPARα activators. In addition, local application of PPARα activators also accelerated the recovery of barrier function after acute barrier damage.^16^ In particular, supplementation with PPARα agonist WY14643 (50 μM) improved homeostasis and barrier function in filaggrin‐deficient skin models by normalizing the free fatty acid profile, underscoring the potential of PPAR agonists in the treatment for filaggrin‐related skin diseases.^17^

Compared with the model group, the boundary between the four layers of the experimental group and positive control group was clearer, the living cell layer was more closely arranged, the integrity of the SC was significantly improved, and the damage of vacuole and living cell layer was significantly reduced. The result in experimental group indicating enhanced skin barrier functionality following cream treatment (Figure 1A).

Effects of investigational cream treatment on expression levels of FLG, LOR in SLS‐induced 3D human epidermis model.

The expression levels of FLG and LOR in the skin model were measured via immunofluorescence (Figure 1). The results showed that after SLS stimulation, the expression levels of FLG and LOR in the model group were significantly lower than those in the control group. Meanwhile, the levels of these proteins in were significantly increased in experimental group and positive control compared to the model group (Figure 1B), the relative content of FLG and LOR in the experimental group was significantly increased by 7.4‐fold and six‐fold (*p* < 0.01). These data indicate that the cream containing C‐xyloside protects SLS‐induced skin model from damage by upregulating FLG and LOR protein expression.

### 3.2 Effect of investigational cream on histomorphology and Ki‐67 in UV‐induced ex‐vivo human skin

UV rays act as aggressors that can lead to a significant reduction in the thickness of living cells in the epidermis, inducing changes in the content of the ECM and contributing to the appearance of aging in the skin.^18^ To investigate the anti‐wrinkle efficacy of cream on human skin, we used UV irradiation of ex‐vivo human skin.

**FIGURE 2:**
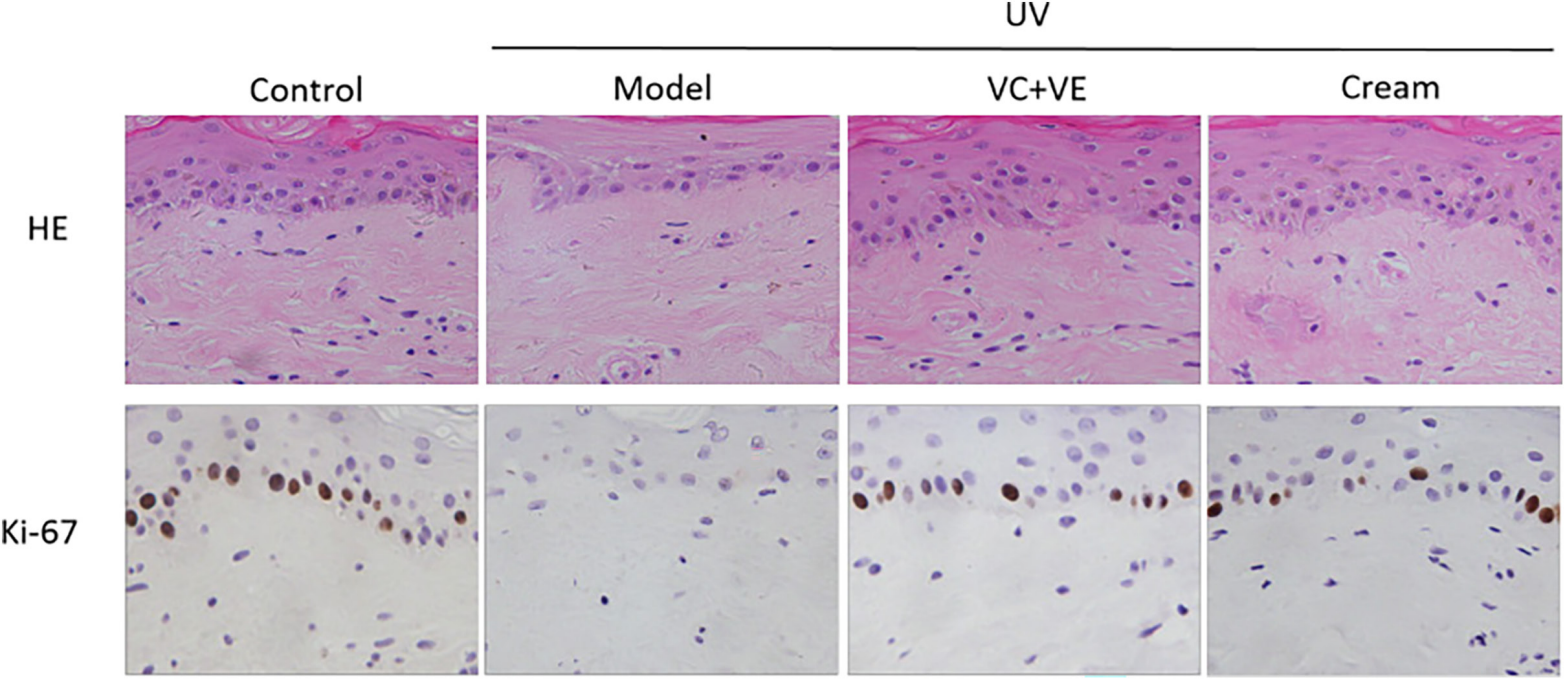
Effects of sample cream treatment on UV‐induced ex‐vivo human skin as assessed via HE staining and Immunohistochemical staining. Compared to the control group, the model group induced by UV irradiation showed a significant decrease in the thickness of epidermal living cells and the density of dermal fibroblasts. However, the thickness of epidermal living cells and dermal fibroblast density of the skin treated with the cream significantly increased compared with the model group, VC (100 μg/mL) and VE (7 μg/mL) were used as the positive control. Compared with the control group, Ki‐67 positive cell was significantly reduced under UV irradiation, and significantly increased under the sample cream and positive control.

Histomorphological changes observed by HE staining analysis. Compared with the control group, the thickness of epidermal living cells and the density of dermal fibroblasts decreased significantly in the UV stimulated group, while in the positive control group and cream group the epidermal living cell thickness and dermal fibroblast density of ex‐vivo human skin increased significantly. Vitamins C and E are potent antioxidants, which effective for the treatment of photoaging.^19^ The result indicated an improvement in skin histomorphology following the sample cream treatment. (Figure 2 HE).

Epidermal regeneration is based on low‐proliferating, self‐maintaining basal stem cells that provide transport expanding cells that give rise to post‐mitotic keratinocytes in 3–5 cell divisions that move up and differentiate into the SC.^20^ The protein Ki‐67 is predominantly localized in the nucleus and exhibits heightened expression levels during cellular division. A direct correlation can be observed between the extent of Ki‐67 positivity and cell proliferation activity.^21^ Hence, the impact of the sample cream on UV‐induced skin cell proliferation can be assessed through Ki‐67 expression (Figure 2). The immunohistochemical results showed that Ki‐67 positive staining cells in the model group decreased significantly, indicating damage to the cells caused by UV irradiation. However, the Ki‐67 positive staining cells in UV‐stimulated skin increased significantly after the use of positive controls. The sample cream treatment group also exhibited significant increases in Ki‐67 positive staining cells, indicating its potential to enhance cellular proliferation in UV‐damaged ex‐vivo human skin.

### 3.3 Effect of investigational cream on collagen synthesis in UV‐induced ex‐vivo human skin

**FIGURE 3:**
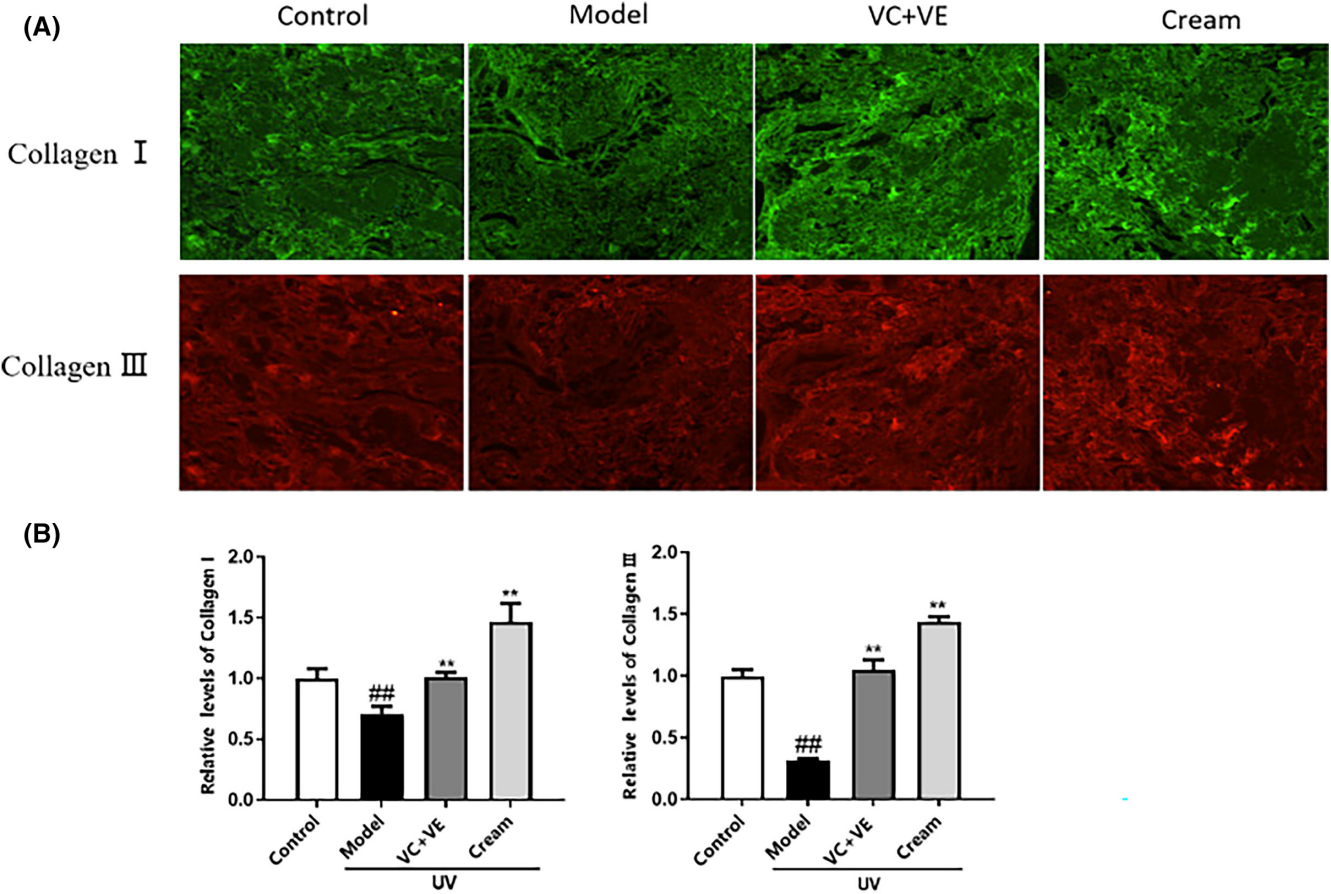
The effect of cream treatment on UV‐induced collagen expression in ex‐vivo human skin was evaluated by immunofluorescence staining. (A) The green fluorescence represents collagen I, and the red fluorescence represents collagen III. The fluorescent signal of collagen I and collagen III of the model group was significantly decreased compared with the blank control group, and the fluorescent signal of collagen I and collagen III was significantly increased compared with the model group after the use of the cream containing C‐xyloside. (B) The relative contents of collagen I and collagen III in each group. Values are expressed as means ± SD (*n* = 3). ^###^ *p* < 0.01 vs. the blank control group; **p < 0.01 vs. the model group.

The immunofluorescence images revealed a significant reduction in the levels of COL I and COL III in the ex‐vivo human skin following UV irradiation, compared to the control group (Figure 3A). Furthermore, treatment with the positive control resulted in a significant increase in protein content of COL I and COL III, as well as an elevated level of COL I and COL III after sample cream treatment (*p* < 0.01), the relative contents of COL I and III were increased by 1.0 and 3.5 times respectively compared with model group (Figure 3B). These findings demonstrate that the sample cream containing C‐xyloside effectively inhibits UV‐induced degradation of COL I and COL III, and increase the content of COL I and III in the dermis after UV irradiation.

**FIGURE 4:**
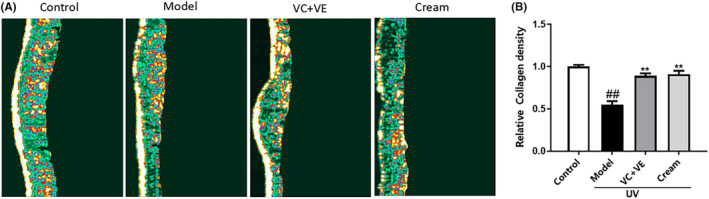
Effect of cream treatment on UV‐induced collagen density in ex‐vivo human skin was evaluated by skin ultrasound. (A) Compared to the control group, the model group exhibited a reduction in highlighted areas, indicating a decrease in collagen density. In contrast, both the sample cream and positive control resulted in an increase in highlighted areas compared to the model group, suggesting enhanced collagen density. (B) The relative collagen density in each group. Values are expressed as means ± SD (*n* = 3). ^###^ *p* < 0.01 vs. the blank control group; **p < 0.01 vs. the UV irradiation group.

Collagen density was measured by skin ultrasound. The higher the collagen density in the dermis the more highlighted areas in the images. Compared with the control group, the dermal highlight area in the model group was significantly reduced, indicating a decrease in collagen density (Figure 4A). Conversely, compared to model group, both the experimental and positive control groups demonstrated a substantial increase in dermal highlight area indicating a significantly increase in collagen density. Specifically, the relative collagen density in the experimental group increased by 0.65 times (Figure 4B), indicating that the cream containing C‐xyloside could prevent the reduction of collagen density induced by UV irradiation in ex‐vivo human skin. This effect may be attributed to its ability to promote synthesis of COL I and COL III.

### 3.4 The repair efficacy of the investigational cream on the facial skin of the volunteers

The epidermis of the skin forms an effective barrier against harmful stimuli from the outside, while acting as a selective permeable membrane to help keep properly moisture in the body.^22^ The SC is the outermost layer of the epidermis. Hydration of SC is very important for its barrier function and cosmetic properties. SC trans‐epidermal water loss (TEWL) and skin water content are two key parameters for SC characterization.^23^ TEWL, the total noneccrine sweat water evaporating from a given area of epidermis over time, it represents the integrity of the skin barrier. Water homeostasis in the epidermis is essential for the normal function of the skin and SC hydration. It is a determinant of skin appearance, mechanical properties, barrier function, and metabolism.

**FIGURE 5:**
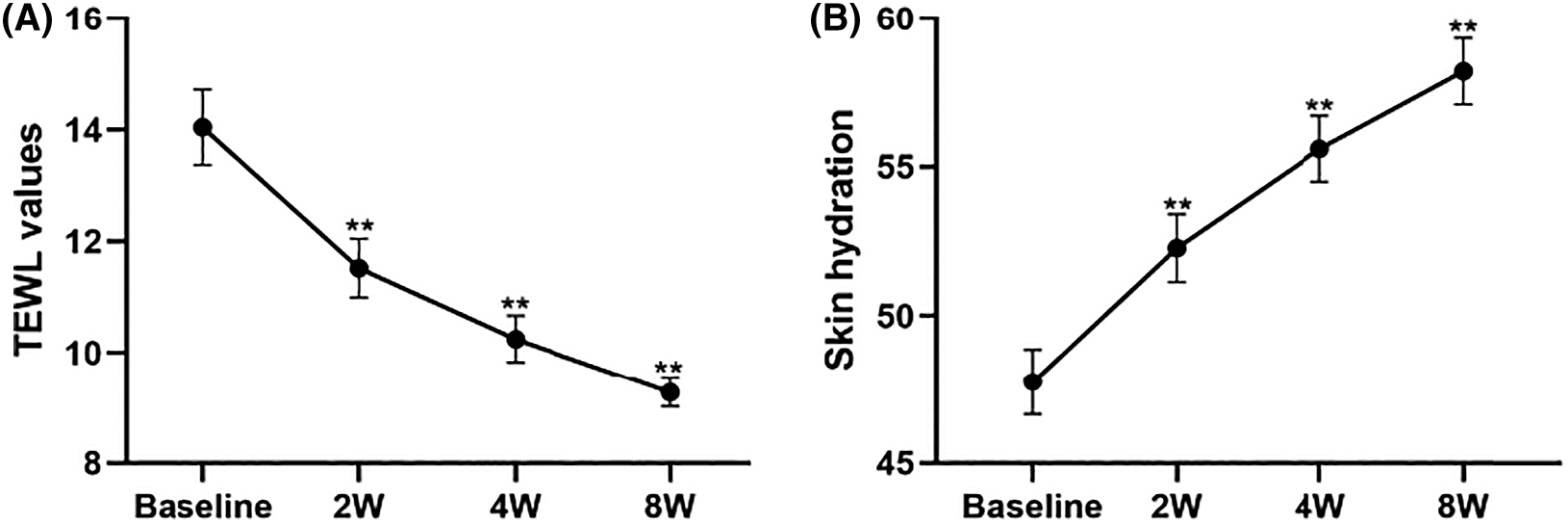
The changes of TEWL values (A) and skin hydration (B) values were measured after 2, 4, and 8 weeks (2 W, 4 W, 8 W) of cream application. The line graph illustrates the values of TEWL and skin hydration. Values are expressed as means ± sem (*n* = 49), and compared with the control group, significance is represented as *, *p* < 0.05 is indicated by *, and *p* < 0.01 is indicated by **.

In this study, we elucidate the impact of investigational cream on the skin barrier through assessment of skin TEWL and skin hydration levels. After using the investigational cream for 2, 4 and 8 weeks, TEWL values significantly decreased from 14.04 ± 0.68 to 11.52 ± 0.53 (−17.92%, *p* < 0.05), 10.25 ± 0.42 (−26.99%, *p* < 0.05) and 9.27 ± 0.26 (−33.94%, *p* < 0.05) respectively. The skin moisture content increased from 47.75 ± 1.11 to 52.27 ± 1.15 (+9.47%, *p* < 0.05), 55.60 ± 1.10 (+16.44%, *p* < 0.05) and 58.20 ± 1.12 (+21.90%, *p* < 0.05) respectively, indicating the significant improvement of skin barrier function after application of investigational cream (Figure 5).

### 3.5 The antiaging efficacy of the investigational cream

**FIGURE 6:**
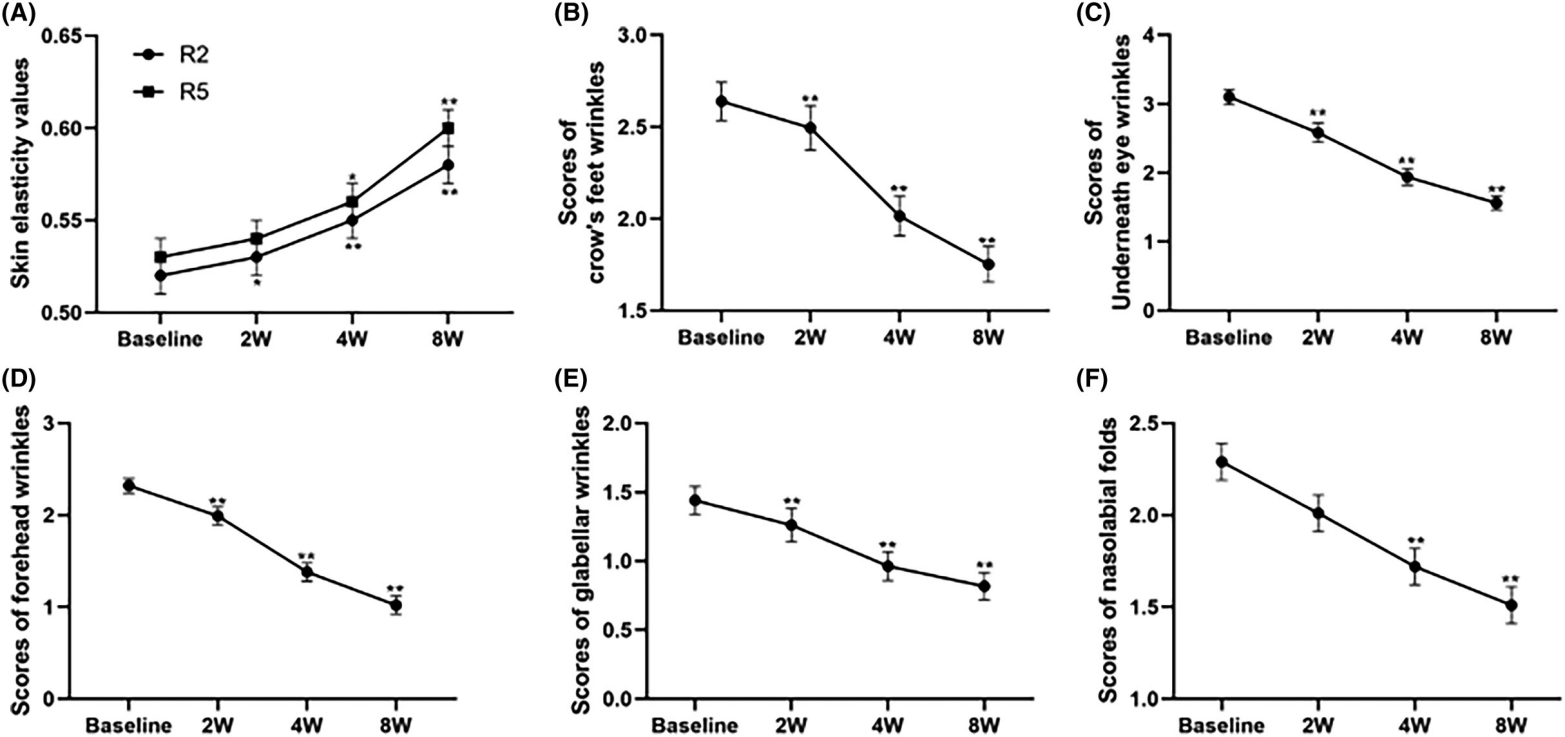
The changes in skin elasticity values and scores of facial wrinkles scores were assessed after 2, 4, and 8 weeks (2 W, 4 W, 8 W) of cream application. The line graph illustrates the value of skin elasticity, and scores of crow's feet wrinkles, underneath eye wrinkles, forehead wrinkles, glabellar wrinkles, and nasolabial folds, while the numerical value denotes its rate of change. Values are expressed as Mean ± SEM (*n* = 49), and compared with the control group, significance is represented as *, *p* < 0.05 is indicated by *, and *p* < 0.01 is indicated by **.

Skin elasticity were measured by a noninvasive skin elasticity meter (Cutometer® Dual MPA 580 (Courage + Khazaka)) at T0, T 2 week, T 4 week, and T8 week. The gross skin elasticity (R2) and net elasticity (R5) of skin were calculated as a ratio of maximum recovery over maximum deformation and that of immediate recovery over immediate deformation respectively. These known measures of skin elasticity were used to assess the effect of investigational cream on the tensile properties of treated skin. The closer the value is to 1, the more elastic is the skin. R2 (gross skin elasticity) values showed significant increase from the baseline value of 0.52 ± 0.01 to 0.58 ± 0.01 after 8 weeks' application (Figure 6; *p* = 0.000), R5 (net elasticity) exhibited significant improvement from 0.53 ± 0.01 to 0.60 ± 0.01 (Figure 6; *p* = 0.000), demonstrated a significantly improvement of the facial elasticity of volunteers after 8 weeks' investigational cream application. The forehead wrinkles, glabellar wrinkles, crow's feet wrinkles, underneath eye wrinkles and nasolabial folds were assessed by dermatologist based on Skin Aging Atlas.^12^

The results demonstrated a significant reduction in crow's feet wrinkles, underneath‐eye wrinkles, forehead wrinkles, and glabellar wrinkles scores after 2, 4, and 8 weeks of cream application (*p* < 0.05, Figure 6 A–E). Nasolabial folds also exhibited similar trends; however, no statistically significant difference was observed at the 2‐week mark (*p* < 0.05, Figure 6F). Overall, the cream effectively improved facial wrinkles among the subjects, particularly after an 8‐week treatment.

**FIGURE 7:**
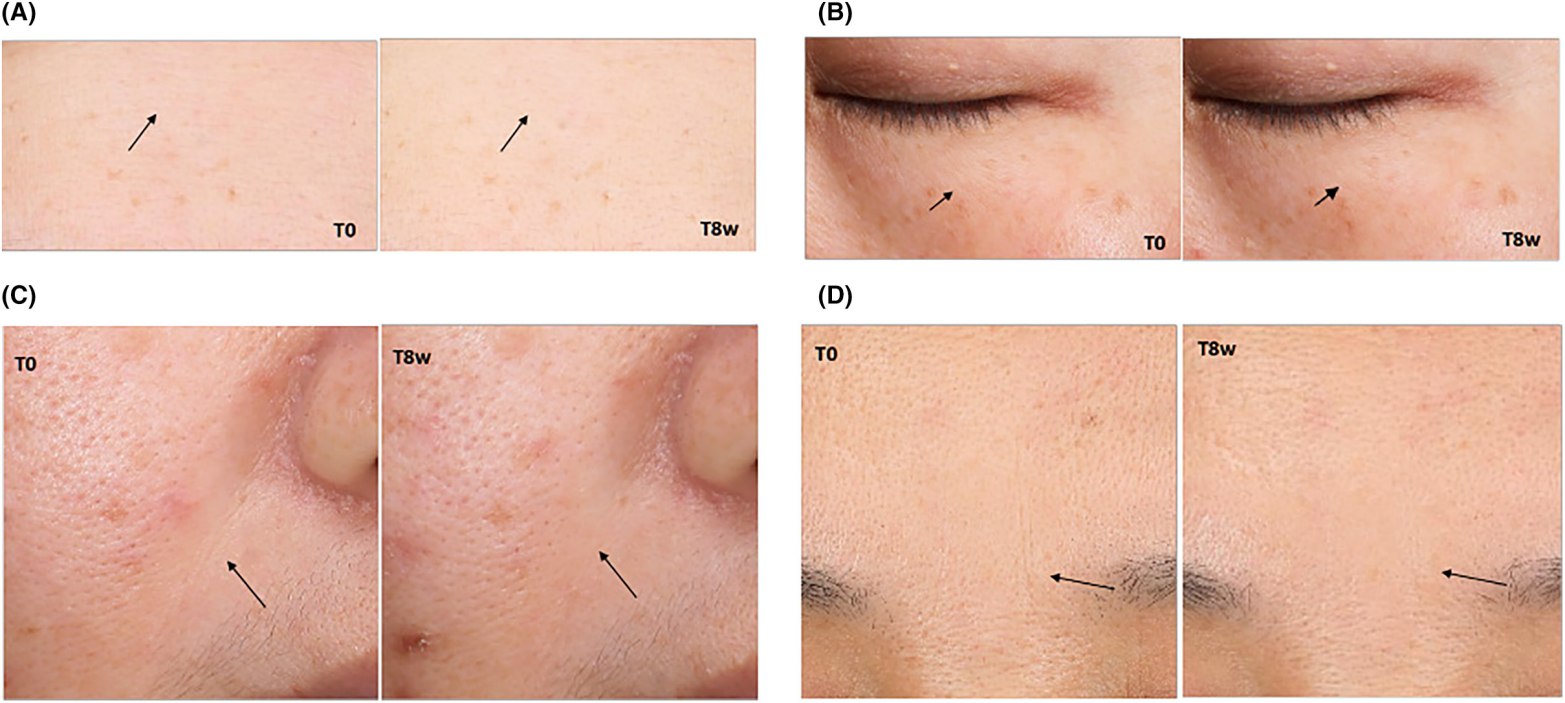
Representative images taken by VISIA 7 of subjects at baseline (T0) and at T8 weeks (T8W) on forehead wrinkles(A), crow's feet wrinkles (B), underneath‐eye wrinkles (B), nasolabial folds (C) and glabellar wrinkles (D). The wrinkles score was graded by dermatologist according to Skin Aging Atlas.^12^

The images (Figure 7; before and after treatment) shown here are average case of the results that have been obtained with investigational cream treatment for 8 weeks.

### 3.6 The repair efficacy of the investigational cream based on volunteers' tape stripping

**FIGURE 8:**
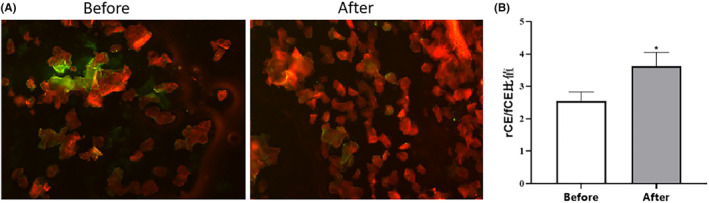
Lipid and IVL expression in facial keratinocytes of volunteers based on tape sampling. (A) Fluorescent and immunofluorescence staining results of keratinocyte lipids (red) and IVL (green) before and after sample cream usage for 4 weeks. (B) Changes in keratinocyte (rCE)/(fCE) ratio of volunteers before and after using the sample cream. Lipid (red) standard rigid corneocyte envelope (rCE), IVL (green) stands for fragile corneocyte envelope (fCE). The value presented as Mean ± SEM, *n* = 30, *p* < 0.05.

The mature cornified envelope (CE) exhibits high hydrophobicity attributed to its lipid envelope and readily stains with lipophilic Nile red; in contrast, the immature CE displays reduced hydrophobicity due to decreased lipid synthesis and incomplete envelope formation, making it more susceptible to binding with anti‐IVL protein antibodies and staining by their associated fluorescent dye. Therefore, evaluating CE maturity based on staining can serve as an effective approach for assessing skin barrier damage. In this study the fluorescence signal of IVL (green) represents the fragile corneocyte envelope (fCE), while the fluorescence signal of lipids (red) indicates the rigid corneocyte envelope (rCE).^24^ A higher ratio of rCE to fCE signifies a greater maturity of CE and an enhanced skin barrier function. The fluorescence staining results revealed that the IVL fluorescence signal was attenuated, and the lipid fluorescence signal was intensified following application of the sample cream (Figure 8A), indicating a more mature CE in the volunteers after treatment. Based on the statistical results (Figure 8B), the average rCE/fCE value of the participants exhibited a significant increase after using the sample cream for 4 weeks compared to before usage (*p* < 0.05, *n* = 30). This date suggests that the CE maturity of the volunteers significantly improved following application of the sample cream, thereby indicating enhanced skin barrier function.

## 4 DISCUSSION

The investigational cream was composed of water, glycerin, propylene glycol, pentanediol, xanthan gum, sodium polyacrylate, butyrospermum parkii (shea) butter, myristyl myristate, C‐xyloside as the active substance were formulated. Glycerin, propylene glycol, pentanediol could be increase cuticle hydration, keep the skin moist, as well as bactericidal and promote transdermal penetration of active substances. xanthan gum and sodium polyacrylate increase cuticle hydration, and also act as emulsifiers and thickeners, giving the cream its sticky shape. butyrospermum parkii (shea) butter and myristyl myristate as the oil phase components in the cream formula give the cream shape and good moisturizing effect. These basic ingredients ensure the basic form and skin feel of the cream, but there are no literature reports on antiaging and barrier repair. C‐xyloside has been fully studied in antiaging, so C‐xyloside is the active ingredient that plays a role in this cream.

Clinical evaluations demonstrated a significant improvement of the skin wrinkles and improved skin condition in subjects who applied the investigational cream mainly containing C‐xyloside, which is similar to previous results.^10^ This study innovatively used the tape stripping method to analyze the effect of investigational creams on barrier differentiation. The results indicated that the cream improved the maturity of cuticle differentiation, which had a potential regulatory effect on barrier differentiation, the results of 3D human epidermis skin model confirmed this hypothesis. The cream effectively repaired SLS‐induced skin injury by up‐regulating the expression of barrier differentiation‐related proteins FLG and LOR. These skin beneficial results may be related to the promotion of glycosaminoglycans(GAGs) synthesis by C‐xyloside. GAGs form an integral part of the skin and make up 0.1%–0.3% total weight. It is reported that dermatan sulfate promoted the proliferation of human dermal fibroblasts (HDF) and the protein expression of COL I, COL III, and elastin.^25^ The cream mainly containing C‐xyloside, promote the synthesis of glycosaminoglycan, significantly increased the content of COL I, COL III and dermal density in ex‐vivo human skin induced by UV irradiation, these results were similar to those of dermatan sulfate induced HDF.

The skin barrier is mainly composed of the epidermal layer, which prevents the loss of water and solutes while acting as a basic protective barrier against the entry of pathogens, irritants and UV rays into the body, thus maintaining homeostasis.^26^ Skin barrier dysfunction caused by endogenous or exogenous factors can lead to various disorders such as xerosis cutis, atopic dermatitis and ichthyoses.^27^ The aging of the skin is often accompanied by a decline in the function of the skin barrier.

The epidermal terminal differentiation forms the skin barrier, through a complex physiological process where the “brick‐mortar” structure of the SC is ultimately formed. Epidermal terminal differentiation is accompanied by the expression of a series of differentiation markers such as FLG, LOR, IVL. FLG aggregates keratin filaments to form keratin networks that bind to the CEs, causing the keratinocytes to collapse into flattened corneocytes. This complex network contributes to the physical strength of the skin. FLG also provides natural moisturizing factors that play an important role in skin barrier functions. FLG insufficiency or absence leads to skin disorders such as AD and ichthyosis vulgaris.^28^ LOR is a terminally differentiating structural protein comprising more than 70% of the CE. It contributes to the protective barrier function of the SC. LOR is an epidermal terminal differentiated structural protein, accounting for more than 70% of the CE. It facilitates the barrier function of the SC. LOR is widely distributed in the surface granular cells in adult epidermis, LOR deficiency or absence has been reported in several diseases exhibiting epidermal barrier dysfunction (generically known as ichthyoses).^29^ IVL also one of the CE proteins synthesized by keratinocyte, showed reduced expression in the lesional skin of atopic dermatitis patients.^30^ It was reported that IVL might be the preferred substrate to which the intercellular lipids(mainly ceramides), are covalently attached to form the outer surface of the CE.^31^ Our study showed that the sample cream significantly promote FLG, LOR protein expression of 3D human epidermis skin model in SLS injury, indicated that the cream containing C‐xyloside promoted the SC differentiation of the injured epidermis. The results of tape stripping demonstrated that the cream effectively facilitated differentiation and maturation of the SC. These results suggest that the cream containing C‐xyloside repairing the barrier function of the skin by promoting the maturation of the SC.

As basal cell proliferation is a prerequisite for epidermal differentiation, maintaining normal proliferative activity is essential for preserving the proper structure and function of the epidermis. When a cell enters mitosis, the chromosomes undergo a series of significant structural changes known as chromosome condensation. A protein sheath called the perichromosome layer (PCL) is present on the outer surface of individual chromosomes, it contains about one‐third of the protein mass of mitotic chromosomes. Ki‐67 is one of the earliest proteins associated with this structure and remained on it until telophase. Some studies have found that Ki‐67 is necessary for PCL formation in humans. Acute Ki‐67 depletion can affect the progression of cell division.^32^

The results of the ex‐vivo human skin showed that Ki‐67 positive cells were mainly distributed in the basal layer of the epidermis. After UV stimulation, a decrease in epidermal thickness was observed along with a reduction in the number of Ki‐67 positive cells, suggesting an inhibitory effect of UV irradiation on cell proliferation within the ex‐vivo human skin. However, after applying the sample cream containing C‐xyloside, both the thickness of the epidermal layer and the number of Ki‐67 positive cells increased in ex‐vivo human skin, indicating that it promoted cell proliferation and increased epidermal thickness.

When the skin is exposed to UV's oxidative stress occurs in the skin, activating cell surface receptors and leading to degradation of the ECM. Receptors activate MAP‐kinases p38, JNK (c‐Jun amino‐terminal kinase), and ERK (extracellular signal‐regulated kinases), respectively, increasing the protein expression of transcription factor AP‐1, which is a key component of the MAP‐kinases. Up‐regulates Matrix metalloproteinases (MMP‐1, MMP‐3) causing collagen degradation.^2^ Decrease in ECM such as collagen fibers is the most significant cause of sagging skin and wrinkles.

Collagen fibers in the dermis are comprised of different collagen types, fibrous collagen types I and III are predominant, which comprise more than 90% of collagen and determine skin strength and hardness.^33^ The results of in vitro studies using ex‐vivo human skin demonstrated that the application of the sample cream significantly stimulated the levels of type I and type III collagen within the skin, as well as enhanced collagen density. These findings contribute to the elucidation of the underlying biological mechanisms responsible for the skin antiaging efficacy exhibited by the creams containing C‐xyloside. Cohen^34^ studied the antiaging effects of antiaging gel containing cannabidiol (CBD) and eicosapentaenoic acid (EPA) using UVB‐induced ex‐vivo skin. The author observed that the antiaging gel inhibited the secretion of PGE2 and IL‐8, increase the synthesis of ECM, and the volunteers’ wrinkles significantly improved after using gel. Another article reported that to determine the recovery effect of the night cream following exposure of the ex vivo human skin to UVA, the author applied night cream immediately after exposure. The level of 8‐OHdG were 49% lower and type I collagen were 19% higher in night cream treated ex vivo human skin exposed to UVA than in untreated. Significant improvement in wrinkles of volunteers have also been observed in clinical trials after cream application.^35^ These results showed that ex vivo human skin model has a strong correlation with clinical trials. The ex vivo human skin culture has been repeatedly shown to emulate several key aspects of intact tissue and is an ideal model for studying the mechanisms of wrinkle formation.

Previous studies have not reported the effects of C‐xyloside on skin barrier proteins and dermal collagen. This study contributes to the advancement of basic biological research on C‐xyloside and uncovers a novel mechanism underlying its antiaging effect.

There are limitations in this study due to the single test concentration and sample size. Although this study cannot directly prove whether investigational cream upregates collagen by promoting collagen synthesis or inhibiting collagen degradation, further studies will be needed to explore the specific mechanisms of action to slow down collagen loss.

## 5 CONCLUSION

The cream containing C‐xyloside exhibits improvement of skin wrinkles and enhancement of skin barrier function (reduced TEWL values, increased skin hydration and rCE/fCE value), thus showing anti‐wrinkle and repairing efficacies. These efficacies may be attributed to the fact that the sample cream can increase the expression of skin barrier related proteins LOR and FLG, promote the maturation of CE, enhance COL I and III protein expression and stimulate skin cell proliferation. These findings provide compelling evidence supporting the efficacy of C‐xyloside cream for skin rejuvenation, thus highlighting its application potentiality in skin antiaging.

## AUTHOR CONTRIBUTIONS

Shanshan Zang, Juanjuan Chen and Jie Qiu designed the experiments and wrote the paper. Cyril Chevalier, Ji Zhang, Shumei Li, Hequn Wang and Jing Li performed the experiments. Yangdong Chen, Hongling Xu, Le Sheng and Zhiming Zhang analyzed the data.

## CONFLICT OF INTEREST STATEMENT

All the authors are employees of L'Oréal (China).

## ETHICS STATEMENT

The ex vivo and volunteers tape stripping experiments were conducted with the approval of Internal Ethical Commission(ethic approval GDLL2022001, BXLL2022036). The protocol and test conditions of the clinical section were conducted with the approval of the Shanghai Ethics Committee For Clinical Research (ethic approval SECCR2021‐207‐02), All participants gave their informed consent to participate in the study.

## Data Availability

The raw data that support the findings of this study are available from the corresponding author upon reasonable request.
